# Examination of validity of identifying congenital heart disease from hospital discharge data without a gold standard: Using a data linkage approach

**DOI:** 10.1111/ppe.12976

**Published:** 2023-03-29

**Authors:** Wen‐Qiang He, Natasha Nassar, Francisco J. Schneuer, Samantha J. Lain, Congenital Heart Disease Synergy Study group, Sally L. Dunwoodie, David Winlaw, Eleni Giannoulatou, Edwin Kirk, Gavin Chapman, Gillian Blue, Gary Sholler

**Affiliations:** ^1^ Child Population and Translational Health Research, Children's Hospital at Westmead Clinical School, Faculty of Medicine and Health University of Sydney Sydney New South Wales Australia

**Keywords:** accuracy, capture–recapture, congenital heart disease, prevalence, validation

## Abstract

**Background:**

Administrative health data has been used extensively to examine congenital heart disease (CHD). However, the accuracy and completeness of these data must be assessed.

**Objectives:**

To use data linkage of multiple administrative data sources to examine the validity of identifying CHD cases recorded in hospital discharge data.

**Methods:**

We identified all liveborn infants born 2013–2017 in New South Wales, Australia with a CHD diagnosis up to age one, recorded in hospital discharge data. Using record linkage to multiple data sources, the diagnosis of CHD was compared with five reference standards: (i) multiple hospital admissions containing CHD diagnosis; (ii) receiving a cardiac procedure; (iii) CHD diagnosis in the Register of Congenital Conditions; (iv) cardiac‐related outpatient health service recorded; and/or (v) cardiac‐related cause of death. Positive predictive values (PPV) comparing CHD diagnosis with the reference standards were estimated by CHD severity and for specific phenotypes.

**Results:**

Of 485,239 liveborn infants, there were 4043 infants with a CHD diagnosis identified in hospital discharge data (8.3 per 1000 live births). The PPV for any CHD identified in any of the five methods was 62.8% (95% confidence interval [CI] 60.9, 64.8), with PPV higher for severe CHD at 94.1% (95% CI 88.2, 100). Infant characteristics associated with higher PPVs included lower birthweight, presence of a syndrome or non‐cardiac congenital anomaly, born to mothers aged <20 years and residing in disadvantaged areas.

**Conclusion:**

Using data linkage of multiple datasets is a novel and cost‐effective method to examine the validity of CHD diagnoses recorded in one dataset. These results can be incorporated into bias analyses in future studies of CHD.

**Synopsis**

**Study question**

Are hospital discharge data a valid data source to identify cases of congenital heart defects for epidemiological research?

**What is already known**

Administrative health data is widely used for research examining congenital anomalies, but it is important data quality and completeness are assessed. Medical record review is the gold standard to validate administrative data; however, it is expensive and time consuming.

**What this study adds**

Data linkage of hospital discharge data with congenital anomaly register, death and outpatient data can be used to examine the validity of recorded diagnoses of congenital heart disease with positive predictive values of up to 94% for severe phenotypes. These multiple data sources also enable identification of various clinical and infant characteristics for bias analyses.

## 1 BACKGROUND

The use of “big data” for health research has grown exponentially over the past few decades. The availability and population reach of administrative data ensure they are a cost‐effective resource for research. However, as these data are collected for non‐research purposes, there have been questions raised regarding their quality and completeness.^1^ Some researchers may avoid the use of routinely collected data for research due to these inaccuracies, and others may accept the data as complete and accurate without further examination.^2^ For optimal use, the potential limitations of the data must be acknowledged, explored and addressed to ensure any biases are minimised.

The gold standard for assessing the accuracy of administrative data is a comparison of these against information recorded in medical records. However, this approach is costly and time consuming. Data linkage of individual records from multiple routinely collected datasets can be used as a method to examine validity of recorded diagnosis.^3^ Data linkage can also be used to minimise underascertainment of conditions in one data source, as different individuals may interact with different health services.^4^

Congenital heart disease (CHD) is the most common congenital anomaly diagnosed in infants worldwide. A recent systematic review of 260 studies reported a global prevalence of 8.2 per 1000 live births from 1970 to 2017.^5^ About half of these estimated CHD prevalence based on data from a congenital anomaly registry. But in settings without a CHD registry, it is necessary to use other population‐based data sources to ascertain the true number and rate of CHD. Hospital discharge data routinely collects information on all inpatient admissions from each hospital, including comprehensive data on clinical diagnoses and related procedures. However, the accuracy and completeness of diagnosis information collected may not be as complete as those data collected by disease‐specific surveillance programs. Previously, it was shown that 93% of congenital anomalies diagnoses recorded in a register were matched to hospital discharge data,^6^ but its accuracy for CHD diagnoses was not determined.

The aim of this study was to use data linkage of multiple administrative health data sources to examine the validity of hospital discharge data to ascertain cases of CHD and its phenotypes.

## 2 METHODS

### 2.1 Study population and data sources

We identified all live births born between January 2013 and December 2017 in New South Wales (NSW), Australia with a diagnosis of a CHD up to 1 year of age recorded in hospital admission data. The Australian health system is a hybrid model whereby government covers the cost of public hospital services, however, purchased private health insurance pays for private hospitals or specialists working in private practice.

The study population was identified by using record linkage of NSW Perinatal Data Collection (PDC – identified all livebirths) and the NSW Admitted Patient Data Collection (APDC – identified CHD diagnosis). The PDC is a population‐based surveillance of all births in NSW of at least 400 grams birth weight or 20 weeks gestation. Each PDC record includes information about both the infant and mother. The APDC records all hospital discharge data from public and private hospitals in NSW (see Supplementary information). All diagnoses are coded based on the 10th revision of the International Classification of Diseases, Australian Modification (ICD‐10‐AM) with a principal diagnosis and up to 50 fields recorded. The APDC records all inpatient procedures and interventions with a principal procedure and up to 50 fields, coded according to the Australian Classification of Health Interventions (ACHI). Individual records from each of the data sources were linked by the NSW Centre for Health Record Linkage using probabilistic linkage methods with a range of personal identifiers. In this study 99% of records in the PDC linked to an APDC record.

The study population was then linked to three other datasets; NSW Register of Congenital Conditions (RoCC), Non‐Admitted Patient Data Collection (NAP) and the Australian coordinating Registry Cause of Death Unit Record File (COD‐URF) (see Supplementary Information). All datasets were restricted to livebirths of residents of NSW. The RoCC is a statutory data collection that requires, under the NSW Public Health Act 2010, doctors, hospitals and laboratories to notify the register when congenital conditions are detected during pregnancy or in an infant up to 1 year of age.^7^ Congenital anomalies in the RoCC are coded using the 9th revision of International Classification of Diseases British Pediatric Association (BPA) Modification Classification. The NAP covers all NSW Health non‐admitted patient services (outpatient services) that have clinical and/or therapeutic content provided or contracted by NSW Health. The COD‐URF is held by the NSW Ministry of Health Secure Analytics for Population Health Research and Intelligence and contains mortality information for deaths occurring in NSW. Underlying and contributing cause of death are recorded by the coroner and/or pathologist or medical practitioner on the death certificate, and coded in COD‐URF using the ICD10. All datasets were available up to 31st December 2018; however, the NAP was only available from 1st January 2015 (see Supplementary information).

### 2.2 Study outcomes and methods to examine the validity of CHD diagnosis in APDC

Infants with CHD were identified from any field of diagnosis in APDC as having at least one record ICD‐10‐AM codes from Q20 to Q26.9. These were then categorised into 17 phenotypes (File S2) based on the hierarchical CHD classification developed by Botto et al 2007^8^ and further modified by Oyen et al 2009.^9^ Infants were further grouped as severe and non‐severe CHD; whereby severe CHD were identified as complex anomalies usually requiring intervention during infancy (File S3).^10^

The diagnosis of CHD in the APDC was examined against any of five reference standards applied in previous studies. Congenital anomaly registers, such as the RoCC, have been used as a gold standard for validating diagnoses of congenital anomalies.^11^ However, the RoCC has been shown to underreport cases of congenital anomalies, with a sensitivity of 75% against a medical record audit.^12^ The reference standards we used to examine validity of CHD diagnoses identified in APDC include matching a CHD diagnosis or a CHD‐related health interaction recorded in addition to or in a different data source than the original CHD APDC diagnosis. This included: (i) multiple hospital admissions (in APDC) containing CHD diagnosis; (ii) receiving a procedure (in APDC) for a cardiac condition (File S4)^13^; (iii) CHD diagnosis recorded in RoCC (File S5)^3^; (iv) cardiac‐related health service recorded in NAP (File S6); and/or (v) cardiac‐related cause of death in the first year of life recorded in COD‐URF (ICD‐10 Q20 to Q26.9).

Two additional characteristics were also evaluated for CHD identified in the APDC: (1)CHD diagnosis recorded in the birth hospital admission, (2) and whether CHD was captured in either the principal diagnosis or the first two (i.e., top 3) diagnosis fields. CHD identified during antenatal screening should be recorded in the birth admission, and the principal diagnosis is mainly responsible for hospital admission. Findings suggest that health conditions recorded in the first few diagnosis fields are often the reason for and more reliable component of the admission record.^14^

Clinical and socio‐demographic characteristics of infants with CHD were also identified to examine differences across data sources. This included, infant sex, plurality, gestational age, birthweight, maternal age, hospital type at birth (public or private), socio‐economic disadvantage, remoteness of residence at birth, any maternal smoking during pregnancy, maternal pre‐existing diabetes, gestational diabetes, maternal pre‐existing hypertension and gestational hypertension, which were identified form the PDC. CHD severity, hospital type where CHD diagnosis recorded (tertiary, non‐tertiary), and presence of syndrome or non‐cardiac congenital anomaly were identified from APDC. The presence of a syndrome was identified using the ICD10‐AM codes (Q87, Q90‐Q99 and U88.2)^15^ and non‐cardiac congenital anomaly defined as infants who had a diagnosis recorded as ICD10‐AM Q‐code for a congenital anomaly other than codes for CHD and syndrome listed as above. Statistical Local Area of residence was obtained from the PDC to identify area of residence at birth. The remoteness of mother's residence was classified using the Accessibility/Remoteness Index of Australia and categorised into three groups (major city, inner regional, outer regional/rural or remote).^16^ Mother's residence was also used to categorise their Socio‐Economic Index for Areas in quintiles from most disadvantaged (Q1) to most advantaged (Q5).^17^

### 2.3 Statistical analysis

To examine data accuracy, the number and percentage of children with a recorded diagnosis of CHD identified from APDC and by each of the five prespecified reference standards were described; overall, by severe and non‐severe and CHD phenotypes. The number of CHD cases in APDC that were also recorded in at least one of the five reference standards was summarised and used to calculate the positive predictive value (PPV) of CHD diagnosis. PPV was defined as the proportion of infants with CHD diagnosis in APDC that were identified by one or more of the five reference standards. For CHD phenotypes, the PPV was calculated against any CHD diagnosis recorded in one of the reference standards, and an additional analysis examined exact match of phenotype group in RoCC with kappa statistics calculated. Clinical and socio‐demographic characteristics associated with validated CHD diagnosis was examined using standardised mean difference. The proportion of CHD diagnoses recorded in the birth admission and recorded in the first 3 diagnosis fields were calculated and were then combined with proportion of CHD cases matched to the five reference standards.

To evaluate CHD cases missed by the APDC, additional CHD cases of any CHD and severe CHD identified by RoCC and/or COD‐URF were numerated. NAP data does not have diagnosis information so additional cases could not be identified from NAP. SAS v9.4 was used for analysis.

### 2.4 Missing data

The following demographic variables were missing <0.5% of data; gestational age, maternal age, maternal smoking and maternal residence. As missing data is less than 5%, no adjustment for missing data was performed.

## 3 RESULTS

**FIGURE 1:**
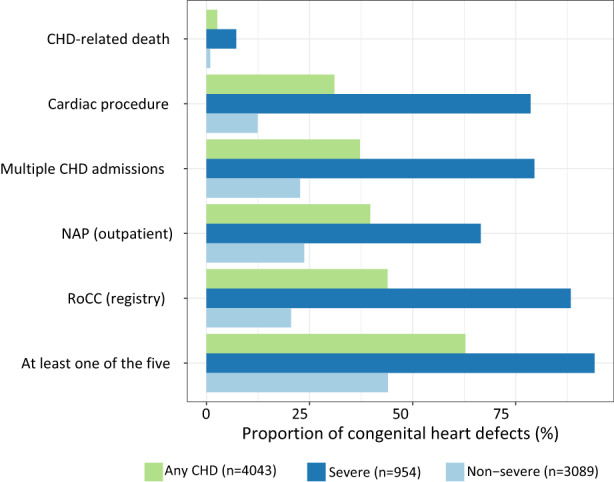
Proportion of congenital heart disease (CHD) identified from hospital discharge data that is supported by another method/data source overall and by severity. APDC top 3 refers to the principal diagnosis and the first two diagnosis of the secondary diagnosis. APDC, admitted patient data collection; CHD, congenital heart disease; NAP, non‐admitted patient; RoCC, registry of congenital condition.

From January 2013 to December 2017, there were 485,239 liveborn infants in NSW recorded in PDC. After excluding records with missing information on gestational age, maternal smoking, maternal age, and maternal residence, 484,806 infants remained in the analysis. There were 4043 infants with a CHD diagnosis identified in the APDC, giving a prevalence of 8.3 per 1000 live births (4043/484,806) and a prevalence of severe CHD of 2.0 per 1000 live births (954/484,806) (Table 1). Of all cases of CHD, 12.5% and 31.7% of them also had a diagnosis of a syndrome or non‐cardiac congenital anomaly, respectively. Of all infants identified with a CHD in the APDC, 2.6% had a CHD‐related death record, 31% had a cardiac procedure recorded, 37.2% had more than one hospital admission for the CHD, 39.7% attended a cardiac‐related outpatient clinic (NAP), and 43.9% had a record in the congenital anomaly register (RoCC). The PPV for a diagnosis of any CHD in the APDC compared with any of the five reference standards was 62.8% (95% CI 60.9, 64.8), with PPV higher for severe CHD at 94.1% (95% CI 88.2, 100) compared to non‐severe CHD at 53.2 (95% CI 51.3, 55.0). The percentage of severe CHD identified by each of the five reference standards was also higher than for non‐severe CHD (Table 1; Figure 1). The PPV for most CHD phenotypes was greater than 75% when compared against any recorded CHD diagnosis, except for non‐severe and unspecified phenotypes; isolated atrial septal defect (ASD), isolated ventricular septal defect (VSD), isolated patent ductus ateriosis (PDA) at term, unspecified CHD and other CHD (Table 1). When comparing the exact phenotype recorded in APDC against RoCC, the percentage of matched phenotypes was lower than the PPVs (Table S1).

**TABLE 1:** Percentage of children with congenital heart disease identified from hospital records (APDC) and supported and validated by other methods/ data sources for overall and CHD phenotypes.

Congenital heart disease (CHD) phenotypes	APDC CHD diagnosis	CHD‐related death *N* (%)	Cardiac procedure *N* (%)	Multiple CHD admissions *N* (%)	NAP (outpatient) *N* (%)	ROCC (congenital anomaly) *N* (%)	At least one the five *N* (%)	PPV (95% CI)
Any CHD	4043	105 (2.6)	1255 (31.0)	1504 (37.2)	1604 (39.7)	1775 (43.9)	2541 (62.8)	62.8 (60.9, 64.8)
Severe CHD	954	69 (7.2)	750 (78.6)	758 (79.5)	634 (66.5)	842 (88.3)	898 (94.1)	94.1 (88.2, 100)
Non‐severe CHD	3089	36 (1.2)	505 (16.3)	746 (24.2)	970 (31.4)	933 (30.2)	1643 (53.2)	53.2 (51.3, 55.0)
Severe phenotype^a^								
Heterotaxia	94	8 (8.5)	40 (42.6)	54 (57.4)	45 (47.9)	64 (68.1)	72 (76.6)	76.6 (61.9, 92.0)
Conotruncal anomaly	714	29 (4.1)	565 (79.1)	573 (80.3)	485 (67.9)	631 (88.4)	677 (94.8)	94.8 (87.9, 100)
AVSD	133	13 (9.8)	104 (78.2)	103 (77.4)	90 (67.7)	116 (87.2)	127 (95.5)	95.5 (79.3, 100)
APVR	76	8 (10.5)	58 (76.3)	56 (73.7)	46 (60.5)	67 (88.2)	71 (93.4)	93.4 (72.5, 100)
LVOTO	294	36 (12.2)	207 (70.4)	222 (75.5)	182 (61.9)	246 (83.7)	274 (93.2)	93.2 (82.6, 100)
RVOTO	339	9 (2.7)	153 (45.1)	212 (62.5)	195 (57.5)	220 (64.9)	282 (83.2)	83.2 (74.4, 92.0)
Complex anomalies	29	‐	24 (82.8)	25 (86.2)	24 (82.8)	26 (89.7)	26.0 (89.7)	89.7 (57.2, 100)
Conotruncal+AVSD	30	6 (20.0)	26 (86.7)	29 (96.7)	22 (73.3)	29 (96.7)	30 (100.0)	100 (64.4, 100)
Septal+LVOTO	175	8 (4.6)	150 (85.7)	151 (86.3)	125 (71.4)	161 (92.0)	169 (96.6)	96.6 (82.3, 100)
Septal+RVOTO	180	‐	120 (66.7)	139 (77.2)	127 (70.6)	150 (83.3)	169 (93.9)	93.9 (80.2, 100)
ASD + VSD	250	‐	103 (41.2)	113 (45.2)	112 (44.8)	161 (64.4)	202 (80.8)	80.8 (70.8, 90.8)
Non‐Severe phenotype^a^								
ASD	621	‐	53 (8.5)	109 (17.6)	173 (27.9)	136 (21.9)	298 (48.0)	48.0 (44.3, 51.7)
VSD	918	6 (0.7)	70 (7.6)	137 (14.9)	239 (26.0)	227 (24.7)	429 (46.7)	46.7 (43.7, 49.7)
PDA, isolated at term	307	‐	38 (12.4)	23 (7.5)	51 (16.6)	18 (5.9)	82 (26.7)	26.7 (23.8, 29.6)
Unspecified phenotype								
Unspecified CHD	60	‐	‐	‐	11 (18.3)	6 (10.0)	22 (36.7)	36.7 (27.5, 45.8)
Other CHD	524	13 (2.5)	88 (16.8)	147 (28.1)	173 (33)	138 (26.3)	280 (53.4)	53.4 (48.9, 58.0)

Abbreviations: ASD, atrial septal defect; APVR, anomalous pulmonary venous return; AVSD, atrioventricular septal defect; CHD, congenital heart disease; LVOTO, left ventricular outflow tract obstruction; NAP, non‐admitted patient; PDA, patent ductus arteriosus; ROCC, registry of congenital condition; RVOTO, right ventricular outflow tract obstruction; VSD, ventricular septal defect. Cell numbers less than 5 not presented.

^a^ Total of “Severe” and “Non‐severe” phenotype groups do not add up to “Severe CHD” and “Non‐severe CHD” groups^10^ as definitions used do not perfectly match up with Botto classification.^8^

Almost 70% of CHD diagnoses were recorded in the birth admission and almost three quarters (73.1%) were recorded in the top 3 diagnosis fields (Table S2). When including CHD recorded in the birth admission or in the first three diagnosis fields as a method to examine validity of CHD, the proportion of CHD cases matched in the APDC increased to 94.4% (Table S2).

When examining the characteristics associated with having a CHD diagnosis supported by a reference standard, a higher proportion of infants with severe CHD were validated than non‐severe CHD (94.1% vs 53.2%) (Table 2). Other infant clinical and socio‐demographic characteristics associated with a higher proportion of CHD diagnosis supported by a reference standard included; lower birth weight, presence of a syndrome or non‐cardiac congenital anomaly, those born in public hospital or admitted to a tertiary hospital and those born to mothers residing in more disadvantaged areas (Table 2). A lower proportion of infants with mothers aged 19 years or younger had a CHD diagnosis supported by a reference standard compared to those born to mothers aged above 20 years.

**TABLE 2:** Clinical and socio‐demographic characteristics of infants with congenital heart disease (CHD) identified from hospital admission records (APDC) up to 1 year of age and supported and validated by at least one of the five methods reference standards.

Clinical and socio‐demographic characteristics	APDC CHD diagnosis *N* (%)	Severe CHD *N* (%)	CHD diagnosis supported and validated *N* (%)	CHD diagnosis not validated supported *N* (%)	SMD
Overall	4043	954	2541 (62.8)	1502 (37.2)	
Severe CHD					0.87
No	3089 (76.4)	‐	1643 (64.7)	1446 (96.3)	
Yes	954 (23.5)	954	898 (35.3)	56 (3.7)	
Sex					0.04
Male	2135 (52.8)	558 (58.5)	1359 (53.5)	776 (51.7)	
Female	1908 (47.2)	396 (41.5)	1182 (46.5)	726 (48.3)	
Plurality					0.04
Single	3747 (92.7)	901 (94.4)	2365 (93.1)	1382 (92.0)	
Twin or higher	296 (7.4)	53 (5.6)	176 (6.9)	120 (8.0)	
Gestational age (weeks)					0.03
≤32	513 (12.7)	50 (5.2)	327 (12.9)	186 (12.4)	
33–36	583 (14.4)	101 (10.6)	358 (14.1)	225 (15.0)	
≥37	2947 (72.9)	803 (84.2)	1856 (73.0	1091 (72.6)	
Birthweight (g)					0.12
<1500	420 (10.4)	43 (4.5)	272 (10.7)	148 (9.9)	
1500–2499	659 (16.3)	131 (13.87)	443 (17.54)	216 (14.4)	
> = 2500	2953 (73.2)	773 (81.60)	1816 (71.58)	1137 (75.7)	
Congenital syndrome					0.43
No	3536 (87.5)	771 (80.8)	2098 (82.6)	1438 (95.7)	
Yes	507 (12.5)	183 (19.2)	443 (17.4)	64 (4.3)	
Other congenital anomaly					0.41
No	2061 (51)	404 (42.3)	1222 (48.1)	1020 (67.9)	
Yes	1982 (49)	550 (57.7)	1319 (51.9)	482 (32.1)	
Maternal age (years)					0.11
≤19	122 (3.0)	35 (3.7)	66 (2.6)	56 (3.7)	
20–24	523 (12.9)	118 (12.4)	354 (13.9)	169 (11.3)	
25–29	1025 (25.4)	237 (24.8)	661 (26.0)	364 (24.2)	
30–34	1289 (31.9)	294 (30.8)	794 (31.2)	495 (33.0)	
35–39	805 (19.9)	200 (21)	493 (19.4)	312 (20.8)	
≥40	279 (6.9)	70 (7.3)	173 (6.8)	106 (7.1)	
Maternal residential location					0.01
Major city	3114 (77.078.5)	716 (75.177.4))	1960 (77.178.6)	1154 (78.36.8)	
Inner regional	669 (16.95)	163 (17.61)	419 (16.85)	250 (17.06.6)	
Outer regional or more	184 (4.6)	46 (5.04.8)	114 (4.65)	70 (4.7)	
Socio‐economic status					0.12
Q1 (most disadvantage)	797 (19.79)	195 (21.10.4)	516 (20.37)	275 (18.73)	
Q2	770 (19.06)	190 (19.920.5)	521 (20.95)	255 (17.30)	
Q3	940 (23.320.4)	186 (19.520.1)	494 (19.84)	314 (21.30.9)	
Q4	744 (18.45)	164 (17.27)	438 (17.62)	294 (19.96)	
Q5	716 (17.721.7)	190 (19.920.5)	524 (21.00.6)	336 (22.85)	
Hospital of birth					0.12
Private	461 (11.4)	74 (7.8)	254 (10.0)	207 (13.8)	
Public	3582 (88.6)	880 (92.2)	2287 (90.0)	1295 (86.2)	
Hospital of CHD admission					0.16
Non‐tertiary	1344 (33.2)	241 (25.3)	775 (30.5)	569 (37.9)	
Tertiary	1827 (45.2)	523 (54.8)	1189 (46.8)	638 (42.5)	
Missing	872 (21.6)	190 (19.9)	577 (22.7)	295 (19.6)	
Maternal smoking					0.01
No	3553 (88.57.9)	858 (89.990.5)	2232 (87.888.5)	1321 (88.7.9)	
Yes	460 (11.54)	90 (9.54)	291 (11.5)	169 (11.3)	
Maternal pre‐existing diabetes					0.07
No	3925 (97.1)	935 (98.0)	2479 (97.6)	1446 (96.3)	
Yes	118 (2.9)	19 (2.0)	62 (2.4)	56 (3.7)	
Gestational diabetes					0.07
No	3603 (89.1)	838 (87.8)	2245 (88.4)	1358 (90.4)	
Yes	440 (10.9)	116 (12.2)	296 (11.6)	144 (9.6)	
Maternal pre‐existing hypertension					0.05
No	3981 (98.5)	941 (98.6)	2496 (98.2)	1485 (98.9)	
Yes	62 (1.5)	13 (1.4)	45 (1.8)	17 (101)	
Gestational hypertension					0.01
No	3776 (93.4)	901 (94.4)	2371 (93.3)	1405 (93.5)	
Yes	267 (6.6)	53 (5.6)	170 (6.7)	97 (6.5)	

Abbreviations: CHD, congenital heart defect; SMD, standardised mean difference. Variables with missing data: for any CHD, 11 missing birthweight, 76 missing maternal residential location and socio‐economic status, 30 missing maternal smoking. For severe CHD, 7 missing birthweight, 29 missing maternal residential location and socio‐economic status and 6 missing maternal smoking.

**FIGURE 2:**
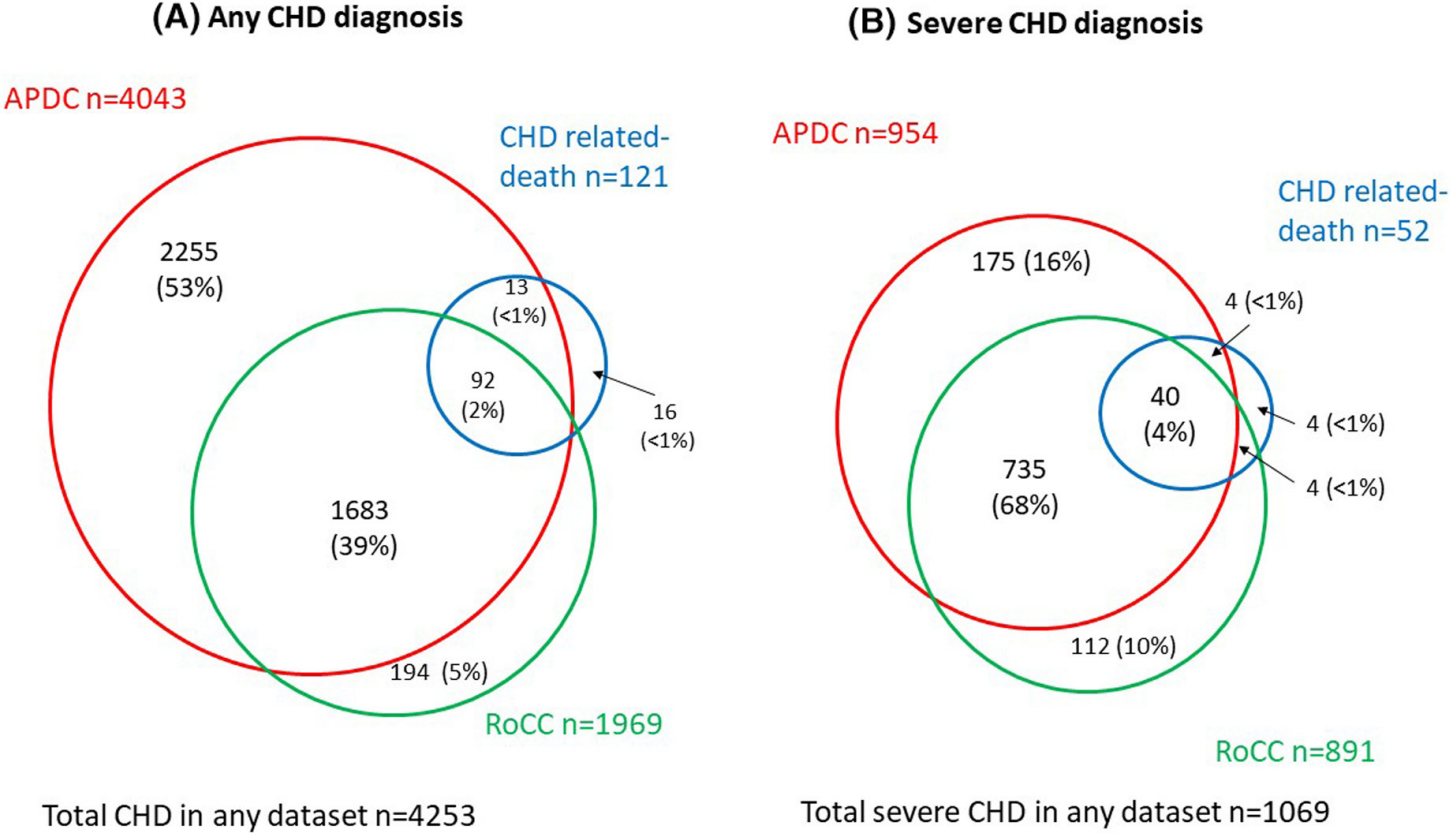
Proportion and overlap of any and severe diagnosis of congenital heart disease identified from 3 different data sources; hospitalisation records (APDC), CHD‐related death records and register of congenital anomaly (RoCC).

Further to the 4043 CHD cases identified from APDC, there were 194 additional CHD cases ascertained from the RoCC and 16 from registered deaths resulting in a total of 4253 CHD cases (Figure 2). For severe CHD identified, in addition to the 954 cases identified in the APDC, an additional 112 cases were recorded in the RoCC and four from deaths data (Figure 2).

## 4 COMMENT

### 4.1 Principal findings

In this study, using data linkage between hospital discharge records and other administrative health datasets, we examined the validity of using hospital data to identify CHD diagnoses and ascertained the number of CHD cases captured in other routinely collected data sources. After examining validity of CHD diagnosis by established methods, a diagnosis of any CHD in the hospital records had a PPV of 62.8%, while a diagnosis of severe CHD a PPV of 94.1% and non‐severe CHD of 53.2%. Observed characteristics associated with CHD diagnosis supported by another data source/method included infants having other syndromes or diagnosed with other anomalies.

#### 4.1.1 Strengths of the study

The main strength of this study was the use of data linkage of multiple datasets to examine the validity of hospital discharge data for identifying CHD amongst infants. For studies that measure disease frequency or burden of CHD and require identification of the whole population, hospital discharge data can be used to identify cases of CHD. The results of this study can inform the development of an algorithm using multiple sources to identify “true cases” of CHD that can be used in studies examining causality. This study can also be used to inform bias analysis in future epidemiological studies examining exposures within a cohort of infants with CHD or identifying CHD as an exposure, particularly the differential identification of CHD amongst individuals by maternal age and socio‐economic status. Traditional bias analysis methods use measures of sensitivity; however, PPVs are often the only measure of data quality for “big data” sources and methods of bias correction using PPVs have been developed.^18^

#### 4.1.2 Limitations of the data

The main limitation of our study was the lack of a gold standard (for instance, medical record review) to validate CHD diagnosis in APDC, although registries such as the RoCC have been used as a gold standard in past studies.^11^ Using the five data sources to examine the validity of CHD diagnosis increased the chance of correct identification in infants that interact more with the health system, however, having increased interaction with the health system does not ensure that the diagnosis would be validated against a medical record audit. Moreover, identifying CHD from the birth admission and the first three diagnosis fields resulted in PPVs similar to published results for studies comparing diagnoses to medical records. With the availability of additional data sources, such as physiologic monitoring and medication data, the validation of diagnoses using data linkage methods for outcome measurement and confounding conditions can be improved.

#### 4.1.3 Interpretation

In this study, we have used five different methods/data sources to examine the classification of infants with CHD. While no Australian study has reported the validation of CHD from hospital discharge data against medical records, routinely collected hospital discharge data against neonatal intensive care audit data had shown high PPV (93%) for selected major anomalies in New South Wales,^19^ which is similar to our reposted PPV of severe CHD cases. In our study, the PPVs for the non‐severe CHD phenotypes, such as ASD and VSD, had much lower PPVs, less than 50%. This is expected as, by definition, infants with severe CHD will have received a cardiac procedure, are likely to be admitted more than once to a hospital for CHD and diagnosed in the first year of life ensuring they are captured by the RoCC, thereby cases of severe CHD in the APDC were more likely to be identified as having any CHD in other datasets than those without a severe phenotype. Furthermore, infants with non‐severe CHD may have their anomaly diagnosed at a later age, and those whose CHD diagnosis is late in the first year of life have had a shorter window of time to access health services within the first year.

The diagnosis of children with non‐severe CHD that were not supported by one of the reference standards were not necessarily false positives. This is because milder conditions are less likely to require hospital contact/ tertiary care and may not have had the chance to be captured by the other data sources included in our study. A recent study from Denmark validated the CHD diagnosis of 463 infants identified using ICD codes recorded in hospital data against medical record review. They found an overall PPV of 95.5% for any CHD, with specific PPVs of 68.9% (95% CI 59.6, 77.3) for ASD and 89.8% (95% CI 82.7, 94.6) for VSD.^20^ When we used additional criteria to examine CHD diagnoses in hospital data alone based on CHD diagnoses identified from the birth admission or top three diagnoses fields, the proportion of diagnoses included increased to 88.6% and 90.5% for ASD and VSD, respectively.

This is the first study to examine the validity of recorded CHD diagnosis by phenotype. The method of phenotype classification by Botto et al has been used widely for studies utilising routinely collected data^5^ as they list ICD10 diagnosis codes for each grouping.^8^ The reporting of PPVs specific to CHD phenotypes ensure that this information can be used in studies examining specific CHD phenotypes. This study found the “Other” and “Unspecified” CHD diagnoses had low PPVs, similar to the earlier mentioned Danish validation study (other CHD anomalies had a PPV of 37.0% (95% CI 20.9, 55.8)).^20^ Depending upon the research question, a sensitivity analyses that examines CHD using a stringent definition, such as excluding these nonspecific CHD diagnoses, should also be performed. Identifying those with more than one admission including a CHD diagnosis has been used to ensure rule‐out diagnoses are not captured as CHD cases.^21^

We also found specific infant characteristics were associated with higher likelihood of having a CHD diagnosis supported by a reference standard. This included infants with a syndrome, other congenital anomalies or low birth weight of <1500 g. These findings are not unexpected as infants with these characteristics are more likely to have contact with health services.^22^ Similarly, infants with CHD born in a public hospital and admitted to a tertiary hospital were more likely to have their diagnoses validated in other data sources. The RoCC staff regularly review medical records at the three Children's hospitals in NSW, which are all situated adjacent to tertiary maternity hospitals, meaning those born in a tertiary hospital and admitted to a Children's hospital will more likely be recorded in the RoCC.^12^ This study also found a differences in PPV by maternal age (lower PPV in young mothers) and socio‐economic status (higher PPV in most disadvantaged areas). This may be because these groups have different access to health services, including access to private specialists that are not captured in these datasets. These results suggest that differences in recording in hospital discharge records in the APDC of CHD in certain groups, such as young mothers, needs to be taken into account when identifying cases and estimating prevalence.

By using hospital discharge records alone, the estimated prevalence of CHD was consistent with a multi‐country systematic review (8.2 vs 8.4 per 1000)^5^; however, the review included data from 1980 onward, over 40 years ago. A more recent study of CHD prevalence using congenital anomaly registry data in Western Australia (WA) reported a prevalence of any CHD amongst livebirths of 10.5 per 1000^23^ higher than the prevalence reported using APDC. The rate of severe CHD amongst livebirths reported in WA was the same as this study, 2.0 per 1000 births.^10^, ^23^ Therefore, the use of the APDC alone to estimate the number and prevalence of severe CHD in liveborns appears to be reliable.

## 5 CONCLUSIONS

This study has shown that using data linkage of multiple data sources is a novel and cost‐effective method to examine the validity of using hospital discharge data to identify CHD cases. Although the use of a medical record review is still the “gold standard” for validating routinely collected data, data linkage is a time and cost‐effective alternative. Prior to using “big data” in epidemiological studies, data quality should be examined and results from internal or external validation studies should be incorporated into bias analyses.

## AUTHOR CONTRIBUTIONS

WQ, NN, and SL conceived and designed the study. NN and SL obtained access to the data. WQ performed the analysis. WQ, NN, FS and SL contributed to data interpretation. WQ and SL drafted the initial manuscript. All authors made substantial contributions to the analysis plan and data interpretation and critically revised the manuscript. The Congenital Heart Disease Synergy Study group gained funding for the study.

## FUNDING INFORMATION

This study has been funded as part of an Australian National Health and Medical Research Council (NHMRC) Synergy Grant (APP1181325). NN is funded by the Financial Markets Foundation for Children and NHMRC Investigator Grant (APP1197940).

## CONFLICT OF INTEREST STATEMENT

All authors have no conflicts of interest.

## Supporting information


Appendix S1.

## Data Availability

Deidentified individual participant data will not be made available.

## 1

Congenital Heart Disease Synergy Study group: Sally L. Dunwoodie, David Winlaw, Eleni Giannoulatou, Edwin Kirk, Gavin Chapman, Gillian Blue, Gary Sholler
