# Supplementary material for: Examination of validity of identifying congenital heart disease from hospital discharge data without a gold standard: Using a data linkage approach
Source: Paediatr Perinat Epidemiol. 2023 Mar 29;37(4):303–12. doi: 10.1111/ppe.12976 (PMC10946896; doi:10.1111/ppe.12976)
Supplement: Supplementary file 1 — Appendix S1. [file PPE-37-303-s001.docx]

**Examination of validity of identifying congenital heart disease from hospital discharge data without a gold standard: using a data linkage approach**

**Short title:** Validity of congenital heart disease diagnoses in hospital discharge data

Wen-Qiang He^1^, Natasha Nassar^1^, Francisco J. Schneuer^1^, Samantha J. Lain^1^ and Congenital Heart Disease Synergy Study group*

1. Child Population and Translational Health Research, Children’s Hospital at Westmead Clinical School, Faculty of Medicine and Health, University of Sydney, NSW Australia

*Congenital Heart Disease Synergy Study group: Sally L Dunwoodie, David Winlaw, Eleni Giannoulatou, Edwin Kirk, Gavin Chapman, Gillian Blue, Gary Sholler

Corresponding author:

Samantha Lain

Child Population and Translational Health Research, Children’s Hospital at Westmead Clinical School, University of Sydney

Sydney, NSW, Australia

Email. [samantha.lain@sydney.edu.au](mailto:samantha.lain@sydney.edu.au)

**Supplementary Table 1**: **Percentage of children with congenital heart disease identified from hospital records (APDC) and register of congenital conditions (RoCC) that match by exact CHD phenotype.**

|  | APDC | RoCC | Matched APDC and RoCC (%) | Kappa statistic |
| --- | --- | --- | --- | --- |
| **Any CHD** | 4043 | 1775 | 1775 (43.9) | 0.07 |
| Severe CHD | 954 | 832 | 775 (81.2) | 0.83 |
| Non-severe CHD | 3089 | 943 | 876 (28.4) | 0.14 |
| **Phenotype** |  |  |  |  |
| Heterotaxia | 94 | 43 | 35 (37.2) | 0.50 |
| Conotruncal anomaly | 714 | 625 | 571 (80.0) | 0.83 |
| AVSD | 133 | 122 | 101 (75.9) | 0.79 |
| APVR | 76 | 60 | 48 (63.2) | 0.70 |
| LVOTO | 294 | 243 | 207 (70.4) | 0.76 |
| RVOTO | 339 | 115 | 81 (23.9) | 0.33 |
| Complex anomalies | 29 | 15 | 14 (48.3) | 0.63 |
| Conotruncal+AVSD | 30 | 28 | 20 (66.7) | 0.69 |
| Septal+LVOTO | 175 | 143 | 106 (60.6) | 0.65 |
| Septal+RVOTO | 180 | 91 | 63 (35.0) | 0.45 |
| ASD | 621 | 163 | 117 (18.8) | 0.25 |
| VSD | 918 | 200 | 153 (16.7) | 0.21 |
| ASD+VSD | 250 | 206 | 115 (46.0) | 0.48 |
| PDA, isolated at term | 307 | 15 | 7 (2.3) | 0.04 |
| Unspecified CHD | 60 | 19 | 0 (0) | 0.00 |
| Other CHD | 524 | 169 | 51 (9.7) | 0.09 |

**Supplementary Table 2**: **Percentage of children with congenital heart disease identified from hospital records using additional two criteria, and total number and PPV using these and reference standards from main analysis, overall and CHD phenotypes.**

|  | APDC | Birth admission  N (%) | APDC top 3 diagnoses  N (%) | % CHD cases validated by one of 5 methods, identified in birth admission or in top 3 APDC diagnoses fields  N (%) |
| --- | --- | --- | --- | --- |
| **Any CHD** | 4043 | 2802 (69.3) | 2969 (73.4) | 3812 (94.2) |
| Severe CHD | 954 | 673 (70.5) | 897 (94.0) | 946 (99.2) |
| Non-severe CHD | 3089 | 2129 (68.9) | 2072 (67.1) | 2866 (92.7) |
| **Phenotype** |  |  |  |  |
| Heterotaxia | 94 | 62 (66.0) | 66 (70.2) | 89 (94.7) |
| Conotruncal anomaly | 714 | 495 (69.3) | 664 (93.0) | 711 (99.6) |
| AVSD | 133 | 75 (56.4) | 116 (87.2) | 129 (96.9) |
| APVR | 76 | 36 (47.4) | 61 (80.3) | 76 (100) |
| LVOTO | 294 | 191 (65.0) | 269 (91.5) | 289 (98.2) |
| RVOTO | 339 | 207 (61.1) | 206 (60.8) | 325 (95.8) |
| Complex anomalies | 29 | 21 (72.4) | 23 (79.3) | 29 (100) |
| Conotruncal+AVSD | 30 | 19 (63.3) | 24 (80.0) | 30 (100) |
| Septal+LVOTO | 175 | 89 (50.9) | 126 (72.0) | 175 (100) |
| Septal+RVOTO | 180 | 67 (37.2) | 93 (51.7) | 180 (100) |
| ASD | 621 | 333 (53.6) | 310 (49.9) | 550 (88.6) |
| VSD | 918 | 729 (79.4) | 719 (78.3) | 831 (90.5) |
| ASD+VSD | 250 | 117 (46.8) | 169 (67.6) | 235 (94.0)) |
| PDA, isolated at term | 307 | 223 (72.6) | 236 (76.9) | 276 (89.0) |
| Unspecified CHD | 60 | 47 (78.3) | 53 (88.3) | 54 (90.0) |
| Other CHD | 524 | 359 (68.5) | 318 (60.7) | 451 (86.0) |

Abbreviations: ASD, atrial septal defect; APVR, anomalous pulmonary venous return; AVSD, atrioventricular septal defect ; CHD, congenital heart disease; LVOTO, left ventricular outflow tract obstruction; NAP, non-admitted patient; PDA, patent ductus arteriosus; ROCC, registry of congenital condition;RVOTO, right ventricular outflow tract obstruction; VSD, ventricular septal defect.

**Supplementary Information file 1: Details of included datasets**

Admitted Patient Data Collection (APDC)

The NSW Admitted Patient Data Collection (APDC) records all inpatient separations (discharges, transfers and deaths) from all public, private, psychiatric and repatriation hospitals in NSW, as well as public multi-purpose services, private day procedure centres and public nursing homes.

Reporting to this data collection is a requirement under the Health Service Act 1997 for public hospitals, and the Private Health Facilities Act 2007 and Health Insurance Act 1973' for private hospitals.^1^

NSW hospitals perform formal audits on ICD-10-AM coded data at a local level. Data edits are monitored regularly and consistent errors are identified and rectified by individual hospitals. All NSW public hospital coded data is routinely processed, monitored and validated using Performance Indicators for Coding Quality by the Ministry of Health and disseminated back to the Local Health Districts and individual hospitals.^2^

Register of Congenital Conditions:

The RoCC is a population-based surveillance system established to monitor congenital conditions detected during pregnancy or at birth, or diagnosed in infants up to 1 year of age. Doctors, hospitals and laboratories are required, under the NSW Public Health Act 2010, to notify certain congenital conditions detected during pregnancy or in an infant up to 1 year of age.

There are 3 types of conditions that are reported to the RoCC:

a) conditions that affect the growth, development and health of the baby that are present before birth, such as cleft lip, dislocated hip and problems with the development of the heart, lungs or other organs;

b) conditions due to changes in the number of the baby’s chromosomes, such as Down syndrome; and

c) four conditions due to changes in the baby’s inherited genetic information: cystic fibrosis, phenylketonuria, congenital hypothyroidism and thalassaemia major.

Children with congenital conditions are commonly treated at the 3 children’s hospitals in NSW: The Children’s Hospital at Westmead, Sydney Children’s Hospital at Randwick, and John Hunter Children’s Hospital. Medical records at these hospitals are regularly reviewed by RoCC staff and congenital conditions are therefore validated prior to being entered onto the RoCC database.^3^

Cause of Death – Unit Record File

Details of all registered deaths are forwarded to the Australian Bureau of Statistics (ABS). The ABS then check and code the information. A single code for an underlying cause of death and multiple cause of death codes (ICD-10) for contributing cause of death are applied to each death record where more than one cause contributed to the death.

The cause of death must be certified by the coroner and/or pathologist or medical practitioner. All deaths for which a coronial inquiry is not required must be certified as to cause and date by a registered medical practitioner and the certificate registered by the Registrar of Births, Deaths and Marriages (RBDM) in each State and Territory. Deaths that are referred to a coroner are registered by the coroner at the conclusion of an inquiry into the circumstances of the death.

Non-admitted patient data (NAP):

The Non-Admitted Patient (NAP) Data Collection is a patient unit record level collection that covers all NSW Health non-admitted patient services that have clinical and/or therapeutic content that warrants a note being made in the client / patient’s medical record. The collection covers all services provided by NSW Health directly to clients / patients irrespective of funding source or mode of service delivery, and all services funded by NSW Health from third party providers under contract.

The collection’s core minimum data set covers the characteristics of the client/patient, the request for service, the organisation service provider, the individual service providers, and each service contact (such as modality, setting, financial class, date of service).

**Supplementary file 2. ICD-10-AM codes for phenotypes of congenital heart disease from Botto et al.^4^**

| Phenotype | ICD-10-AM |
| --- | --- |
| 1. Heterotaxia | Q24.0, Q24.1, Q89.3, Q20.6 |
| 1. Conotruncal anomaly | Q20.0, Q25.1A, Q25.2, Q25.3, Q25.4, Q20.3, Q21.3, Q20.1, without Q21.2; (Q21.4, without Q21.1 or Q21.2); (Q22.1 and Q21.0, without Q21.2); (Q25.5 and Q21.0, without Q21.2) |
| 1. Atrioventricular septal defect (AVSD) | (Q21.2, without Q21.3) |
| 1. Anomalous pulmonary venous return (APVR) | Q26.2, Q26.4, Q26.8, Q26.9, without Q21.2; Q26.3, without Q21.2 |
| 1. Left ventricular outflow tract obstruction (LVOTO) | Q23.4, (Q25.1, without Q21.0), Q23.0, Q23.1 |
| 1. Right ventricular outflow tract obstruction (RVOTO) | Q22.1 only, Q22.4, Q22.5, (Q25.5, without Q21.0 or Q21.3), Q25.6 |
| 1. Isolated atrial septal defect (ASD) | Q21.1 only |
| 1. Isolated ventricular septal defect (VSD) | Q21.0 only |
| 1. ASD and VSD | Q21.0 and Q21.1 only |
| 1. Complex defects | Q20.4 |
| 1. Conotruncal defect+AVSD | Q20.0, Q25.1, Q25.2, Q25.3, Q25.4, Q20.3, Q21.3, Q20.1, (Q21.4 without Q21.1), (Q22.1 and Q21.0), (Q25.5 and Q21.0) and Q21.2 |
| 1. Septal defect+LVOTO | (Q21.0 and Q25.1), (Q21.1 and Q21.0 and Q23.0, Q23.1A), (Q21.1 and Q21.0 and Q25.1) |
| 1. Septal defect+RVOTO | Q21.1 and Q22.1, Q21.0 and Q22.1, (Q21.1 and Q21.0 and Q22.1) |
| 1. Isolated patent ductus arteriosus (PDA) in infants born at term | Q25.0 only |
| 1. Isolated PDA in preterm infants | Q25.0 only |
| 1. Unspecified | Q24.9 only |
| 1. All other specified CHDs | from Q20 to Q26.9 apart from the above category |
|  |  |

**Supplementary information file 3. List of severe congenital heart defects and the corresponding ICD-10-AM codes used.** Abbreviation: AVSD, Atrioventricular septal defect; TGA, transposition of the great arteries; VSD, ventricular septal defect.

| Severe diagnosis description | ICD-10-AM |
| --- | --- |
| Functionally univentricular heart including single ventricle, tricuspid valve atresia, aortic atresia, pulmonary valve atresia with intact ventricular septum, double inlet left ventricle, hypoplastic left heart syndrome, hypoplastic right ventricle. | Q20.4, Q22.42, Q25.2, Q22.0 (without Q21.0), Q20.41, Q23.4, Q20.81 |
| TGA | Q20.3 |
| Congenitally corrected TGA | Q20.51 |
| Truncus arteriosus | Q20.0 |
| Interrupted aortic arch | Q25.13 |
| AVSD | Q21.2 |
| Double outlet right ventricle | Q20.1 |
| Coarctation of the aorta | Q25.1 |
| Pulmonary valve atresia with VSD | Q22.0, Q21.0 |
| Tetralogy of Fallot | Q21.3 |
| Total anomalous pulmonary venous return | Q26.2 |
| Heterotaxy or isomerism | Q20.6, Q24.0, Q24.1, Q20.6, Q89.3 |

**Supplementary information file 4. ICD-10-AM codes for cardiac condition related procedure.**

| Procedure descriptions | Procedure codes |
| --- | --- |
| Insertion, removal and replacement of a cardiac pacemaker or electrode | 38253, 38256, 38259, 38278, 38281, 38350, 38353 |
| Procedure on the atrium, cardiac vessels and aorta | 38456, 38457, 38458, 38460, 38464, 38466, 38470, 38473, 38475, 38477, 38480, 38481, 38483, 38485, 38487-38490, 38493, 38497, 38500, 38503-38505, 38507-38509, 38512, 38515, 38518, 38521, 38524, 38530, 38533, 38550, 28553, 38556, 38559, 38562, 38565, 38568, 38571 |
| Procedures on the ventricle | 38615, 38618, 38621, 38624, 38627-00 |
| Procedures on the myocardium | 22075, 38418, 38275, 38577, 38588, 38560, 38650, 90206 |
| Closure of cardiac collateral vessels | 38700.00-38700.03 |
| Percutaneous interventions including angioplasty, atrial septostomy, balloon valvuloplasty, baffle and conduit procedures and procedures on the pulmonary and coronary arteries | 38270, 38300, 38303, 38306, 38309, 38312, 38315, 38318, 38637, 38706, 38715, 38721, 38727, 38733, 38739, 38742, 38745, 38748, 38751, 38754, 38757, 38760, 38763, 38766 |
| Cardiopulmonary bypass | 38588.00, 38600.00, 38603.00, 90225.00, 38627.02, 38653 |

**Supplementary information file 5: Code matched between ICD-10-AM and British Paediatrics Association (BPA) for the diagnosis of congenital heart defect.**

| ICD-10-AM_desription | ICD-10-AM code | BPA description | BPA code |
| --- | --- | --- | --- |
| Common arterial trunk | Q20.0 | Truncus arteriosus | 74500 |
| Double outlet right ventricle | Q20.1 | Double outlet right ventricle | 74513 |
| Double outlet left ventricle | Q20.2 | Double outlet left ventricle | 74514 |
| Discordant ventriculoarterial connection | Q20.3 | Transposition of great vessels - incomplete | 74511 |
| Discordant ventriculoarterial connection | Q20.3 | Transposition of great vessels - corrected | 74512 |
| Discordant ventriculoarterial connection | Q20.3 | D-type transposition of great vessels | 74515 |
| Discordant ventriculoarterial connection | Q20.3 | L-type transposition of great vessels | 74516 |
| Discordant ventriculoarterial connection | Q20.3 | Transposition of great vessels - other specified NEC | 74518 |
| Discordant ventriculoarterial connection | Q20.3 | Transposition of great vessels - NOS | 74519 |
| Transposition of great vessels, complete | Q20.31 | Transposition of great vessels - complete | 74510 |
| Double inlet ventricle | Q20.4 | Common ventricle | 7453 |
| Hypoplastic left ventricle | Q20.82 | Hypoplastic left ventricle | 746880 |
| Ventricular septal defect | Q21.00 | Ventricular septal defect - NOS | 74549 |
| Muscular ventricular septal defect | Q21.01 | Ventricular septal defect - muscular | 74544 |
| Perimembranous ventricular septal defect | Q21.02 | Ventricular septal defect - perimembranous | 74543 |
| Subarterial ventricular septal defect | Q21.03 | Ventricular septal defect - subaortic | 74545 |
| Gerbode defect | Q21.04 | Ventricular septal defect - Gerbode defect | 74542 |
| Ventricular septal defect | Q21.09 | Ventricular septal defect - Eisenmenger's syndrome | 74541 |
| Ventricular septal defect | Q21.09 | Ventricular septal defect - other specified NEC | 74548 |
| Atrial septal defect, unspecified | Q21.10 | Atrial septal defect - NOS | 74559 |
| Patent or persistent foramen ovale | Q21.11 | Patent foramen ovale | 74550 |
| Ostium secundum defect | Q21.13 | Atrial septal defect - ostium secundum defect | 74551 |
| Other specified atrial septal defect | Q21.19 | Atrial septal defect - other specified NEC | 74558 |
| Artrioventricular septal defect | Q21.2 | Common atrium | 74561 |
| Artrioventricular septal defect | Q21.2 | Endocardial cushion defect - other specified NEC | 74568 |
| Ostium primum defect | Q21.21 | Endocardial cushion defect - ostium primum defect | 74560 |
| Incomplete common atrioventricular septal defect | Q21.22 | Common A-V canal - incomplete | 74563 |
| Complete common atrioventricular septal defect | Q21.24 | Common A-V canal type ventricular septal defect - complete | 74562 |
| Tetralogy of Fallot | Q21.3 | Tetralogy of Fallot | 74520 |
| Aortopulmonary septal defect | Q21.4 | Aortic septal defect - aortopulmonary window | 74501 |
| Other congenital malformations of cardiac septa | Q21.8 | Anomaly of cardiac septal closure - NOS | 7459 |
| Pentalogy of Fallot | Q21.83 | Pentalogy of Fallot | 74521 |
| Pulmonary valve atresia | Q22.0 | Atresia of pulmonary valve | 74600 |
| Congenital pulmonary valve stenosis | Q22.1 | Stenosis of pulmonary valve | 74601 |
| Congenital pulmonary valve insufficiency | Q22.2 | Insufficiency of pulmonary valve | 74602 |
| Other congenital malformations of pulmonary valve | Q22.3 | Absence of pulmonary valve | 74604 |
| Other congenital malformations of pulmonary valve | Q22.3 | Hypoplasia or dysplasia of pulmonary valve | 74605 |
| Other congenital malformations of pulmonary valve | Q22.3 | Anomaly of pulmonary valve - other specified NEC | 74608 |
| Other congenital malformations of pulmonary valve | Q22.3 | Anomaly of pulmonary valve - NOS | 74609 |
| Congenital tricuspid stenosis | Q22.41 | Stenosis of tricuspid valve | 74611 |
| Congenital tricuspid atresia | Q22.42 | Atresia of tricuspid valve | 74610 |
| Ebstein's anomaly | Q22.5 | Ebstein's anomaly | 7462 |
| Hypoplastic right heart syndrome | Q22.6 | Hypoplastic right heart | 746884 |
| Congenital tricuspid insufficiency | Q22.81 | Insufficiency of tricuspid valve | 74612 |
| Congenital tricuspid dysplasia | Q22.82 | Hypoplasia or dysplasia of tricuspid valve | 74613 |
| Other specified congenital malformations of tricuspid valve | Q22.89 | Anomaly of tricuspid valve - other specified NEC | 74618 |
| Congenital malformation of tricuspid valve, unspecified | Q22.9 | Anomaly of tricuspid valve - NOS | 74619 |
| Congenital stenosis and atresia of aortic valve | Q23.0 | Stenosis of aortic valve | 74631 |
| Congenital atresia of aortic valve | Q23.02 | Atresia of aortic valve | 74630 |
| Congenital insufficiency of aortic valve | Q23.1 | Insufficiency of aortic valve | 74640 |
| Congenital mitral stenosis | Q23.21 | Stenosis of mitral valve | 74651 |
| Congenital mitral atresia | Q23.22 | Atresia of mitral valve | 74650 |
| Congenital mitral insufficiency | Q23.3 | Insufficiency of mitral valve | 74660 |
| Congenital mitral insufficiency | Q23.3 | Anomaly of mitral valve - NOS | 74669 |
| Congenital mitral insufficiency | Q23.3 | Anomaly of mitral valve - other specified NEC | 74668 |
| Hypoplastic left heart syndrome | Q23.4 | Hypoplastic left heart syndrome | 7467 |
| Hypoplastic left heart syndrome | Q23.4 | Hypoplastic left heart | 746883 |
| Other congenital malformations of aortic and mitral valves | Q23.8 | Anomaly of aortic valve - other specified NEC | 74648 |
| Other congenital malformations of aortic and mitral valves | Q23.8 | Anomaly of aortic valve - NOS | 74649 |
| Congenital aortic valve dysplasia | Q23.81 | Hypoplasia or dysplasia of aortic valve | 74642 |
| Congenital mitral valve dysplasia | Q23.82 | Hypoplasia or dysplasia of mitral valve | 74661 |
| Congenital biscuspid aortic valve | Q23.83 | Bicuspid aortic valve | 74641 |
| Dextrocardia | Q24.0 | Dextrocardia without situs inversus | 74680 |
| Laevocardia | Q24.1 | Levocardia | 74681 |
| Cor triatriatum | Q24.2 | Cor triatriatum | 74682 |
| Pulmonary infundibular stenosis | Q24.3 | Pulmonary infundibular stenosis | 74683 |
| Congenital subaortic stenosis | Q24.4 | Subaortic stenosis | 74632 |
| Malformation of coronary vessels | Q24.5 | Coronary aneurysm | 746881 |
| Malformation of coronary vessels | Q24.5 | Anomaly of coronary artery | 746886 |
| Other specified congenital malformations of heart | Q24.8 | Anomaly of heart - other specified NEC | 746889 |
| Congenital cyanotic heart disease | Q24.83 | Congenital heart disease - cyanotic | 74693 |
| Other specified congenital malformations of heart | Q24.89 | Ectopic heart | 746882 |
| Congenital malformation of heart, unspecified | Q24.9 | Anomaly of heart - NOS | 74699 |
| Patent ductus arteriosus | Q25.0 | Patent ductus arteriosus | 7470 |
| Coarctation of aorta | Q25.1 | Coarctation of aorta - preductal | 74710 |
| Coarctation of aorta | Q25.1 | Coarctation of aorta - postductal | 74711 |
| Coarctation of aorta | Q25.1 | Coarctation of aorta - NOS | 74719 |
| Coarctation of aorta | Q25.1 | Coarctation of aorta - juxtaductal | 74713 |
| Interrupted aortic arch | Q25.13 | Interrupted aortic arch | 74712 |
| Atresia of aorta | Q25.2 | Atresia of aorta | 747201 |
| Supravalvular aortic stenosis | Q25.31 | Supra-aortic stenosis | 74722 |
| Other congenital malformations of aorta | Q25.4 | Vascular ring of aorta - double aortic arch | 74725 |
| Other congenital malformations of aorta | Q25.4 | Aneurysm of aorta | 74727 |
| Other congenital malformations of aorta | Q25.4 | Anomaly of aorta - other specified NEC | 74728 |
| Other congenital malformations of aorta | Q25.4 | Anomaly of aorta - NOS | 74729 |
| Absence of aorta | Q25.41 | Absence of aorta | 747200 |
| Hypoplasia of aorta | Q25.44 | Hypoplasia of aorta | 74721 |
| Persistent aortic arch | Q25.45 | Persistent right aortic arch | 74723 |
| Overriding aorta | Q25.47 | Overriding aorta | 74726 |
| Atresia of pulmonary artery | Q25.5 | Atresia of pulmonary artery with septal defect | 74731 |
| Atresia of pulmonary artery | Q25.5 | Absence or agenesis of pulmonary artery | 747300 |
| Atresia of pulmonary artery | Q25.5 | Atresia of pulmonary artery | 747301 |
| Stenosis of pulmonary artery | Q25.6 | Stenosis of pulmonary artery | 74732 |
| Other congenital malformations of pulmonary artery | Q25.7 | Hypoplasia of pulmonary artery | 74735 |
| Other congenital malformations of pulmonary artery | Q25.7 | Anomaly of pulmonary artery - other specified NEC | 74738 |
| Congenital aneurysm of pulmonary artery | Q25.71 | Aneurysm of pulmonary artery | 74733 |
| Congenital aneurysm of pulmonary artery | Q25.71 | Arteriovenous aneurysm - pulmonary | 74734 |
| Congenital stenosis of vena cava | Q26.0 | Stenosis of vena cava - inferior | 747401 |
| Persistent left superior vena cava | Q26.1 | Persistent left superior vena cava | 74741 |
| Total anomalous pulmonary venous connection | Q26.2 | Total anomalous pulmonary venous return | 74742 |
| Partial anomalous pulmonary venous connection | Q26.3 | Partial anomalous pulmonary venous return - NOS | 747430 |
| Other congenital malformations of great veins | Q26.8 | Anomaly of great veins - other specified NEC | 74748 |
| Scimitar syndrome | Q26.81 | Scimitar syndrome | 747431 |
| Congenital absence and hypoplasia of umbilical artery | Q27.0 | Absence or hypoplasia of umbilical artery | 7475 |
| Congenital renal artery stenosis | Q27.1 | Stenosis of renal artery | 74760 |
| Other congenital malformations of renal artery | Q27.2 | Anomaly of renal artery - other specified NEC | 747619 |
| Peripheral arteriovenous malformation | Q27.3 | Arteriovenous malformation - peripheral | 74762 |
| Other specified congenital malformations of peripheral vascular system | Q27.8 | Anomaly of peripheral arteries - other specified NEC | 74764 |
| Other specified congenital malformations of peripheral vascular system | Q27.8 | Anomaly of peripheral veins - other specified NEC | 74765 |
| Other specified congenital malformations of peripheral vascular system | Q27.8 | Anomaly of peripheral vascular system - other specified NEC | 74768 |
| Arteriovenous malformation of cerebral vessels | Q28.2 | Arteriovenous aneurysm - brain/cerebral vessels | 74780 |
| Other malformations of cerebral vessels | Q28.3 | Anomaly of cerebral vessels - other specified NEC | 74781 |
| Other specified congenital malformations of circulatory system | Q28.8 | Absent ductus arteriosus | 74783 |
| Situs inversus | Q89.30 | Situs inversus - NOS | 75939 |
| Dextrocardia with situs inversus | Q89.31 | Situs inversus (complete) with dextrocardia | 75930 |
| Situs inversus | Q89.33 | Situs inversus abdominis | 75933 |
| Situs inversus | Q89.34 | Situs inversus thoracis | 75932 |

**Supplementary information file 6. NAP cardiac-related health services to be validated as CHD in NAP**

| **Service** | Cardiology |
| --- | --- |
|  | Cardiothoracic surgery |
|  | Cardiac catheterization |
|  | Cardiac rehabilitation |
| **Service Unit** | Clinical measurement-cardiology diagnostic unit |
|  | Cardiology medical consultation unit |
|  | Cardiac rehabilitation allied health/nursing unit |
|  | Pacemaker medical consultation unit |
|  | Angioplasty/angiography procedure unit |
|  | Cardiothoracic medical consultation unit |
|  | Vascular and Interventional radiology |

1. Australian Bureau of Statistics. NSW HEALTH DEPARTMENT, ADMITTED PATIENT DATA COLLECTION. 2007; <https://www.abs.gov.au/AUSSTATS/abs@.nsf/Lookup/1368.1Explanatory%20Notes1452007> Accessed on 27/10/2022.

2. Australian Institute of Health and Welfare. Australian Hospital statistics, 2012-2013. In: Government A, ed*.* Vol Health services series no. 54. Cat. no. HSE 145. Canberra2014.

3. Centre for Epidemiology and Evidence. *Quality and Coverage of the NSW Register of Congenital Conditions Using Admitted Patient Data: A Record Linkage Study.* Sydney: NSW Ministry of Health; 2016.

4. Botto LD, Lin AE, Riehle‐Colarusso T, Malik S, Correa AJBDRPAC, Teratology M. Seeking causes: classifying and evaluating congenital heart defects in etiologic studies. *Birth Defects Research Part A: Clinical and Molecular Teratology.* 2007;79(10):714-727.
